# Laryngeal Penetration and Risk of Aspiration Pneumonia in Children with Dysphagia—A Systematic Review

**DOI:** 10.3390/jcm12124087

**Published:** 2023-06-16

**Authors:** Aamer Imdad, Alice G. Wang, Vaishali Adlakha, Natalie M. Crespo, Jill Merrow, Abigail Smith, Olivia Tsistinas, Emily Tanner-Smith, Rachel Rosen

**Affiliations:** 1 Division of Pediatric Gastroenterology, Hepatology, and Nutrition, SUNY Upstate Medical University, Syracuse, NY 13210, USA; 2 Norton College of Medicine, SUNY Upstate Medical University, Syracuse, NY 13210, USA; wanga@upstate.edu (A.G.W.);; 3 Division of Pediatric Gastroenterology, Department of Pediatrics, McGovern Medical School, UTHealth Houston, Houston, TX 77030, USA; vaishali.a.harne@uth.tmc.edu; 4 Department of Otolaryngology, SUNY Upstate Medical University, Syracuse, NY 13210, USA; 5 Health Science Library, SUNY Upstate Medical University, Syracuse, NY 13210, USAtsistijo@upstate.edu (O.T.); 6 College of Education, University of Oregon, Eugene, OR 97403, USA; etanners@uoregon.edu; 7 Division of Pediatric Gastroenterology, Department of Pediatrics, Boston Children Hospital, Boston, MA 02115, USA; rachel.rosen@childrens.harvard.edu

**Keywords:** child, deglutition disorders, pneumonia, aspiration, larynx, laryngeal penetration, aspiration pneumonia

## Abstract

This study was a systematic review and meta-analysis that assessed the risk of aspiration pneumonia in children with laryngeal penetration or tracheal aspiration via a video-fluoroscopic study (VFSS) and compared the results to those for children with neither condition. Systematic searches were conducted using databases, including PubMed, Cochrane Library, and Web of Science. Meta-analysis was used to obtain summary odds ratios (OR) and 95% confidence intervals (CI). The overall quality of evidence was assessed using the grading of recommendations, assessment, development, and evaluation (GRADE) criteria. In total, 13 studies were conducted with 3159 participants. Combined results from six studies showed that laryngeal penetration on VFSS may be associated with aspiration pneumonia compared to no laryngeal penetration; however, the summary estimate was imprecise and included the possibility of no association (OR 1.44, 95% CI 0.94, 2.19, evidence certainty: low). Data from seven studies showed that tracheal aspiration might be associated with aspiration pneumonia compared to no tracheal aspiration (OR 2.72, 95% CI 1.86, 3.98, evidence certainty: moderate). The association between aspiration pneumonia and laryngeal penetration through VFSS seems to be weaker than that for tracheal aspiration. Prospective cohort studies with clear definitions of laryngeal penetration and that measure clinical and patient reported outcomes are needed to further define the association between laryngeal penetration and aspiration pneumonia.

## 1. Introduction

Approximately 500,000 U.S. children are affected by oropharyngeal dysphagia [1]. Some of the most common causes of oropharyngeal dysphagia include premature birth, developmental delay, neuromuscular disorders, anatomic abnormalities, and cardiopulmonary disease [2]. Clinical evaluation of children with swallowing difficulty includes, but is not limited to, obtaining a medical history, performing a baseline examination of structural and physiologic components of swallowing, and observing interactions with caregivers during oral intake. Additional studies, such as video-fluoroscopic swallow studies (VFSS) and/or fiberoptic endoscopic evaluation of swallowing (FEES), are used to visualize the upper aerodigestive tract [3]. The VFSS, also known as the modified barium swallow study, is considered to be the most objective study employed to evaluate swallowing function in children. 

VFSS provides objective data about the co-ordination of bolus propulsion from the oral cavity to the pharynx and esophagus, as well as information about laryngeal penetration and tracheal aspiration [4]. Laryngeal penetration is defined as the passage of material into the larynx without passing through the vocal cords. In contrast, tracheal aspiration is defined as the passage of material below the vocal cords into the trachea [5], and is associated with increased odds of aspiration pneumonia [6]. While tracheal aspiration is strongly associated with aspiration pneumonia in children [7], the association between laryngeal penetration and aspiration pneumonia remains unclear [8,9]. Some retrospective studies showed an increased incidence of aspiration pneumonia in children with laryngeal penetration [10,11], whereas other studies did not find evidence of this correlation [12,13,14]. We conducted a systematic review and meta-analysis to assess the association between laryngeal penetration and the risk of aspiration pneumonia in children with oropharyngeal dysphagia. 

## 2. Materials and Methods

We followed the Preferred Reporting Items for Systematic Reviews and Meta-Analyses (PRISMA) guidelines when reporting this review [15]. A protocol was developed before the start of the review and published in a peer review journal [16]. The protocol is registered at the International prospective register of systematic reviews (PROSPERO) website (registration number CRD42020222145). The methods are briefly described below. 

*Study type*: Observational studies were included, including cohort and case-control studies. We excluded case series, case reports, and observational studies with no comparison group.

*Population*: The population of interest was pediatric patients (<18 years old) with dysphagia who were previously evaluated using VFSS or FEES. We considered studies that included a mixed population with and without anatomical problems. We also included studies in which the anatomical problem was unknown before VFSS was performed. However, we excluded studies that focused exclusively on patients with a particular anatomical anomaly of the aerodigestive tract (e.g., cleft palate) or a specific medical diagnosis (e.g., myasthenia gravis, post-stroke) because the results from these studies may not be generalizable to the population of interest. 

*Exposure*: Our primary exposure of interest was laryngeal penetration found using VFSS or FEES in pediatric patients with dysphagia. We excluded studies in which VFSS or FEES was not performed. We considered exposure to occur (‘yes’) or not occur (‘no’) (laryngeal penetration vs. no laryngeal penetration for consistency). In addition, we considered the degree of laryngeal penetration based on the 8-point penetration–aspiration scale [5,16].

*Comparison*: We compared the exposure group to patients who had neither penetration nor aspiration on VFSS or FEES [16]. We also compared the findings with those of tracheal aspiration in children. Studies that did not include a comparison group were excluded.

*Outcomes*: The primary outcome measure was the incidence of aspiration pneumonia. We used the WHO definition of pneumonia in children between 2 and 59 months of age as cough and/or difficulty breathing with tachypnea and/or chest indrawing [17]. Secondary outcomes included the incidence of hospitalization, pediatric intensive care unit (PICU) admission, enteral tube requirement, all-cause mortality, weight for age, height for age, BMI for age, changes in feeding practices, and adverse events. 

*Literature Search*: Systematic electronic searches were conducted using multiple electronic databases, including PubMed, EMBASE, Web of Science, CINHAL, Scopus, Cochrane CENTRAL, LILACS, and WHO Global Index Medicus. The last date of the literature search was 11 July 2022. We also searched the Clinicaltrials.gov website to identify ongoing studies. No search restrictions were applied based on language, outcomes, publication status, or publication date. We also searched the reference sections of previously published studies. We employed two librarians on our team, who helped with the literature search. The search strategy was published using a protocol [16]. 

*Selection of studies*: Study selection was completed through a three-stage process. Two authors screened titles to identify potentially eligible studies. Any study determined to be eligible at this step proceeded to the second stage of screening for a full-text review. Finally, any studies retained after the full-text review were moved to the third step of full data extraction [16]. Covidence software was used for screening and data extraction [18]. 

*Data Extraction*: We designed and used a data extraction sheet to collect information from selected studies [16]. Three authors (AW, VA, and NC) independently used data extraction sheets and compared their findings. Any incongruence or question was resolved through discussion with the help of the senior author on the team (AI). We extracted information on the study site, year, population, exposure (laryngeal penetration, tracheal aspiration), comparison, outcomes, risk of bias, and confounding factors [16]. 

The authors of the included studies did not use a uniform definition of pneumonia. Therefore, we assessed the pneumonia definition in each of the included studies to determine whether they followed the WHO definition. If a study did not follow the WHO definition, we noted that and still included data from that study as reported by the authors. If the authors reported the data on pneumonia based on severity (mild, moderate, severe) and these categories were mutually exclusive, we combined these categories to create one outcome (pneumonia, yes or no). 

*Assessment of risk of bias in included studies*: The Cochrane Risk of Bias in Non-Randomized Studies of Interventions (ROBINS-I) tool was used to assess the risk of bias in the included studies. This tool evaluates each study as a non-randomized comparison trial and covers domains through which bias may be introduced. We addressed five risk of bias domains using signaling questions: bias due to confounding, bias in the selection of participants for the study, bias due to missing data, bias in the measurement of outcomes, and bias in selecting the reported result. Each domain received a judgment regarding the risk of bias as low, moderate, serious, or critical. The highest risk of bias in one domain determined a study’s overall risk of bias, regardless of the lower risks in other domains [19].

*Data synthesis*: The findings from all included studies were reported in a narrative synthesis. We also conducted meta-analyses to synthesize evidence across studies quantitatively. Dichotomous outcomes were combined to obtain a summary odds ratio (OR) and reported with corresponding 95% confidence intervals (CIs). Continuous outcomes were combined using the mean difference effect size and reported with their corresponding 95% CIs. We used random effects models to pool effect sizes using the generic inverse variance method of meta-analysis. The most adjusted values from the included studies were used. The software RevMAN [20] was used for statistical analysis.

*Dealing with missing data*: Study attrition was noted during the data extraction. We contacted the authors if data were missing for some cases or if reasons for dropout were not reported. If the data were missing key variables, the authors requested additional data.

*Assessment of heterogeneity*: Heterogeneity was defined as any variability among studies in a systematic review. Clinical heterogeneity is variability among participants, interventions, and outcomes studied; methodological heterogeneity is variability in study design, outcome measurement tools, and risk of bias; and statistical heterogeneity is variability in the intervention effects being evaluated in different studies, which result from clinical or methodological heterogeneity [21]. Statistical heterogeneity was assessed using I2 statistics and χ2 statistics [22]. Low, moderate, and high relative levels of heterogeneity were defined with upper limits of 25%, 50%, and 75% for I2, respectively. Calculated values were considered significant for heterogeneity when the I2 value was >50%, or the *p*-value was <0.1. Subgroup analyses were performed to determine the reasons for statistically significant heterogeneity [16].

*Assessment of reporting bias*: We planned to present funnel plots to assess the small-study bias when a meta-analysis included at least 10 studies. As none of the meta-analyses had at least 10 studies, we did not create funnel plots. 

*Subgroup analysis and investigation of heterogeneity*: We planned a priori for the following subgroup analyses: study population—age (infants < 1 year of age vs. children 1–5 years of age); neurological comorbidity (children with CNS anomalies vs. children with no known CNS anomalies) and syndromic comorbidity (children with a syndromic diagnosis vs. children with no syndromic diagnosis); anatomic anomalies of the airway or gastrointestinal tract (children with anatomical anomalies vs. children with no anatomical anomalies); and exposure (penetration–aspiration scale scores of 0–2 vs. 3–5 vs. 6–8). None of these subgroup analyses could be conducted because of the lack of data available for these subgroups in the included studies. 

*Sensitivity analysis*: We planned to accomplish three goals in the sensitivity analysis: exclude studies with a high overall risk of bias, use a random vs. fixed effects model, and include studies that used the WHO definition of pneumonia vs. non-WHO/non-standard definition of pneumonia. 

*Rating of overall quality of evidence*: We assessed the overall quality of evidence for the association of exposure with an outcome using the Grading of Recommendations Assessment, Development, and Evaluation (GRADE) approach with the GradePro software [23]. This approach identified and assessed the different features that affected the certainty of evidence in a review, including the type of study design, within-study risk of bias, heterogeneity, directness of evidence, risk of publication bias, and precision of effect estimates. The GRADE method results in ratings of certainty of the evidence for an outcome as very low, low, moderate, or high [24]. 

## 3. Results

### 3.1. Literature Search

**Figure 1:**
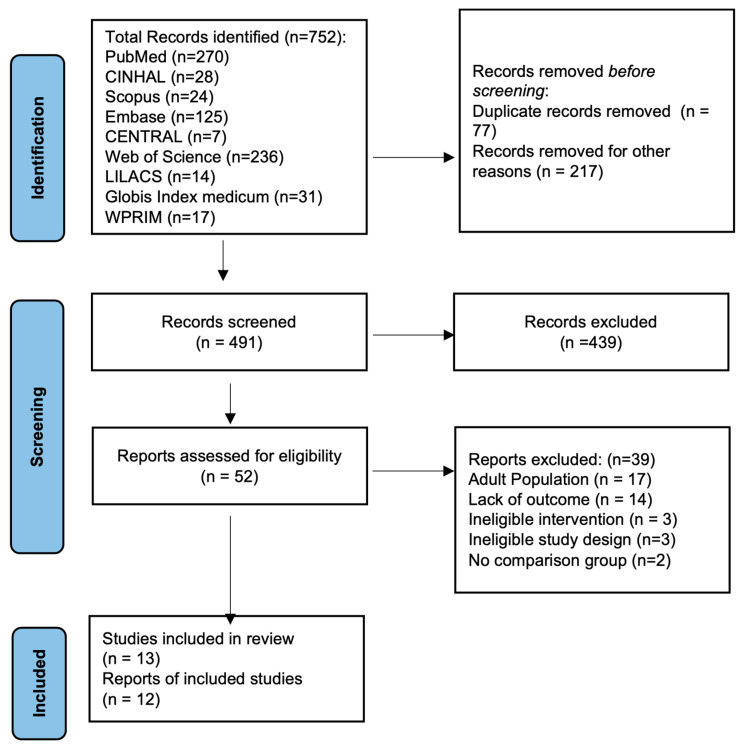
PRISMA flow diagram of study identification. Abbreviations: CINHAL: Cumulative Index to Nursing and Allied Health Literature; LILACS: Literatura Latino-Americana e do Caribe em Ciências da Saúde; WPRIM: Western Pacific Region Index Medicus.

Figure 1 presents the results of the literature search. A total of 803 titles and abstracts were screened. Of these, 54 eligible studies were screened for full-text reviews; ultimately, 13 studies met the inclusion criteria and were included [6,7,11,12,13,25,26,27,28,29,30,31,32]. Thirty nine studies were excluded [33,34,35,36,37,38,39,40,41,42,43,44,45,46,47,48,49,50,51,52,53,54,55,56,57,58,59,60,61,62,63,64,65,66,67,68,69,70,71,72]; the reasons for exclusion are listed in Table S1. The most common reasons for exclusion included studying an adult population, a lack of outcomes studied, a lack of exposure of interest or comparison, and ineligible study design. 

### 3.2. Characteristics of Included Studies

Table 1 presents the characteristics of the included studies.

#### 3.2.1. Country

The included studies were conducted in six different countries, including six studies conducted in the United States [6,13,25,26,28,29] and two studied conducted in each of Australia [7,32], Canada [11,12], Brazil [30], India [31], and South Korea [27]. 

#### 3.2.2. Study Design 

Four studies were case controlled [6,7,11,31], one study was a prospective cohort [32], and eight studies were retrospective cohorts [12,13,25,26,27,28,29,30].

#### 3.2.3. Population

The study population consisted of pediatric patients who were referred for swallowing evaluation. The years of data collection ranged from 1989 to 2018. The median sample size of the included studies was 108 (range: 19–1668). In all 13 studies, there were 1857 males and 1302 females. The majority of patients studied in the included studies were under two years of age. There were 110 patients with known syndromes, the most common of which was Down’s syndrome. 

#### 3.2.4. Exposure

All 13 studies used VFSS to evaluate swallowing function. Seven studies defined laryngeal penetration as the entry of material into the laryngeal vestibule but not below the true vocal cords [4,6,10,11,22,24,27]. Nine studies defined tracheal aspiration as the entry of material below the level of the true vocal cords or into the subglottic airway [4,6,10,11,22,24,25]. Two studies used Rosenbek’s penetration–aspiration scale [30,32].

#### 3.2.5. Comparison

Seven studies compared laryngeal penetration to no laryngeal penetration or tracheal aspiration [6,7,11,12,13,30,31]. Eight studies compared tracheal aspiration to no laryngeal penetration and tracheal aspiration [6,7,12,13,27,30,31,32]. Three studies compared laryngeal penetration to tracheal aspiration [26,28,29].

#### 3.2.6. Risk of Bias in the Included Studies

The risk of bias in the included studies was assessed using the ROBINS-I tool. None of the included studies had a low risk of bias owing to small sample sizes and failure to adjust for potential confounders (Supplementary Table S2). Below, we describe the risk of bias for each analysis. 

### 3.3. Outcomes

We described our results according to three comparisons: ‘laryngeal penetration vs. no laryngeal penetration’, ‘tracheal aspiration vs. no tracheal aspiration’, and ‘laryngeal penetration vs. tracheal aspiration’. All = included studies contributed data to at least one outcome, except for one study [25], in which the data were presented in a way that meant that they could not be meta-analyzed for any of the analyses. 

#### 3.3.1. Laryngeal Penetration vs. No Laryngeal Penetration or Tracheal Aspiration

##### Primary Outcome: Incidence of Pneumonia

**Figure 2:**
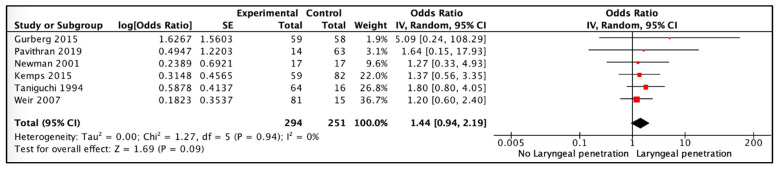
Association of laryngeal penetration vs. no laryngeal penetration with aspiration pneumonia. The figure shows the names of the included studies on the left and the data on the right side with numerical data from each study and summary estimate presented both numerically and graphically. The width of (red) squares in the graphical presentation of the summary estimate on the extreme right indicate magnitude of effect and the lines indicate 95 % CI [6,7,11,12,13,31].

The pooled data from these six studies, with 294 participants with laryngeal penetration and 251 participants with no laryngeal penetration, showed a low certainty of evidence that laryngeal penetration may be associated with increased odds of aspiration pneumonia; however, the confidence interval around the summary estimate was imprecise and included the possibility of no association (OR 1.44, 95% CI, 0.94, 2.19; I2 = 0%; Figure 2). We downgraded the certainty of the evidence due to the risk of bias and imprecision of the summary estimates (Table S3). 

##### Sensitivity Analysis 

A fixed vs. random effects model did not change the results substantially (OR 1.44, 95% CI 0.94, 2.19). A sensitivity analysis excluding two studies [11,13] that were at high risk of bias did not change the magnitude of the summary effect, though it widened its confidence interval (OR 1.42, 95% CI 0.91, 2.21). In a sensitivity analysis based on the definition of pneumonia used by study authors, the one included study [7] that used the pneumonia definition devised by the WHO reported an effect (OR 1.20, 95% CI 0.60, 2.40) similar to the overall estimate presented above. 

##### Secondary Outcomes

One study reported data on enteral tube requirement, including 66 participants with laryngeal penetration and 46 without laryngeal penetration [30]. The results were imprecise and included the possibility of no association (OR 0.77, 95% CI 0.26, 2.29). We downgraded the certainty of evidence owing to the low risk of bias and imprecision of the summary estimate (Table S3). One study [25] that was not included in the meta-analysis reported data on the association between intermediate abnormalities for VFSS (which had laryngeal penetration, pooling, delayed initiation of swallow, and disco-ordinated phases of swallowing) and acute respiratory illness (which included inpatient, outpatient, and emergency department visits for aspiration pneumonia, pneumonia, bronchiolitis, and asthma). The intermediate abnormalities were not associated with an acute respiratory illness irrespective of intervention received, such as thickening of feeds [Hazard ratio (HR) 1.30, 95% CI 0.73–2.29)] or placement of a nasogastric tube (HR 2.15, 95% CI 0.75–6.18). The outcomes of incidence of hospitalization, PICU admission, mortality, weight for age, height for age, BMI for age, and adverse events were not reported in any of the included studies. 

#### 3.3.2. Tracheal Aspiration vs. No Tracheal Aspiration or Laryngeal Penetration 

##### Primary Outcome: Incidence of Pneumonia

**Figure 3:**
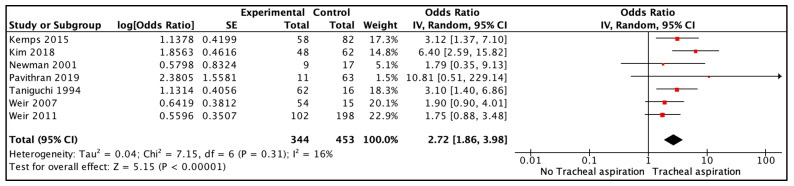
Association of tracheal aspiration vs. no tracheal aspiration with aspiration pneumonia. The figure shows the names of the included studies on the left and the data on the right side with numerical data from each study and summary estimate presented both numerically and graphically. The width of (red) squares in the graphical presentation of the summary estimate on the extreme right indicate magnitude of effect and the lines indicate 95 % CI [6,7,12,13,27,31,32].

Seven studies reported data on tracheal aspiration vs. no laryngeal penetration or tracheal aspiration. The data for aspiration pneumonia included 797 participants in total, with 344 participants with tracheal aspiration and 453 participants without tracheal aspiration. The combined results showed a moderate certainty of evidence that tracheal aspiration found on VFSS might be associated with increased odds of aspiration pneumonia in the exposed group compared to the control group (OR 2.72, 95% CI 1.86, 3.98, I2 = 16%, Figure 3). We downgraded the certainty of evidence owing to the risk of bias in the included studies (Table S4). 

##### Sensitivity Analysis 

The fixed-effects model usage did not change the summary estimates. In the sensitivity analysis excluding the two studies [12,13] with a high risk of bias, the confidence interval around the summary estimate was widened (OR 2.81, 95% CI 1.66, 4.77). When the analysis was restricted to the two studies [7,32] that used the pneumonia definition devised by the WHO, the magnitude of the summary estimate was smaller, and its corresponding confidence interval was wider than the overall estimate presented above (OR 1.82, 95% CI 1.10, 3.01). 

##### Secondary Outcomes

Data on enteral tube requirement included 412 participants in total, with 139 participants with tracheal aspiration and 273 participants without tracheal aspiration [30,32]. The results showed higher odds of having a feeding tube in the exposed group compared to the control group (OR 1.32, 95% CI 0.58, 2.98). We downgraded the certainty of evidence due to the risk of bias and imprecision of the summary estimate (Supplementary Table S3). One study reported a higher risk of hospitalization in patients with tracheal aspiration compared to no laryngeal penetration or tracheal aspiration (RR 1.04, 95% CI 1.01, 1.07) [30]; however, the absolute risk seems very small. One study [25] that was not included in the meta-analysis reported data on the association between oropharyngeal aspiration (that included tracheal aspiration with thin and thick feeds with and without cough) and acute respiratory illness. The study found no association between oropharyngeal aspiration and acute respiratory illness, irrespective of the degree of aspiration or type of intervention offered, such as thickening of feeds or placement of a nasogastric tube. The only significant association was decreased risk of acute respiratory illness when thickened feeds were provided in case of silent aspiration [25]. Incidences of PICU admission, mortality, weight for age, height for age, BMI for age, changes in feeding practices, and adverse events were not reported in any of the included studies.

#### 3.3.3. Tracheal Aspiration vs. Laryngeal Penetration

Two studies reported data on tracheal aspiration vs. laryngeal penetration for aspiration pneumonia; these studies included 45 participants, of whom 20 had tracheal aspiration, and 25 had laryngeal penetration. The results were very imprecise, and no solid conclusion could be drawn from these data (OR 1.45, 95% CI 0.37, 5.73). 

##### Secondary Outcomes

Data on enteral tube requirements included 1717 participants in total, with 808 participants with tracheal aspiration and 909 participants with laryngeal penetration. The results showed higher odds of enteral tube placement in the tracheal aspiration group compared to the laryngeal penetration group (OR 3.65, 95% CI, 2.43, 5.48) [26,28,29]. No other secondary outcomes were reported when comparing tracheal aspiration and laryngeal penetration. 

## 4. Discussion

### 4.1. Summary of Main Results

This systematic review and meta-analysis evaluated the association between laryngeal penetration and tracheal aspiration and the risk of aspiration pneumonia in pediatric patients with dysphagia who are undergoing a video-fluoroscopy study. The pooled analysis suggests that the odds of aspiration pneumonia were lower for laryngeal penetration (OR 1.44, 95% CI 0.94, 2.19) than for tracheal aspiration (OR 2.72, 95% CI 1.86, 3.98) when compared to children with no laryngeal penetration or tracheal aspiration. The analysis of tracheal aspiration vs. laryngeal penetration did not include enough studies to make a conclusive statement. Data were unavailable for several key secondary outcomes, such as hospitalization, intensive care admission, symptom improvement, and mortality.

### 4.2. Quality of Evidence

All included studies were observational studies, which carry an inherent risk of bias due to concerns about selection bias and adjustment for confounding factors. Most of the included studies had small sample sizes, and there were concerns about missing data. Several studies also did not adjust for confounding factors, which increased the risk of bias in summary estimates from these studies (Supplementary Table S2). However, we conducted a sensitivity analysis for the outcome of aspiration pneumonia by excluding studies with a high risk of bias. The direction of the effect did not change, though the summary estimates became imprecise, having a wider confidence interval. We incorporated this concern regarding the risk of bias into the GRADE ratings by downgrading the certainty of the evidence for the association between laryngeal penetration and tracheal aspiration and aspiration pneumonia. We also downgraded for imprecision to compare laryngeal penetration vs. no laryngeal penetration and tracheal aspiration vs. laryngeal penetration for aspiration pneumonia risk, because the 95% CIs around the pooled summary estimate was wide and included the possibility of no association. 

### 4.3. Strengths and Limitations

We followed the standard guidelines of the Cochrane Collaboration for this review. The study’s questions and methods were described in a protocol published in a peer-reviewed journal before the start of the study [16]. The main limitation of the data presented in the included studies was that most data did not report important clinical outcomes beyond aspiration pneumonia, such as hospitalization; intensive care unit admission; long term complication, such as bronchiectasis and bronchopulmonary disease; growth outcomes; quality of life; and mortality. The definition of exposure (laryngeal penetration) varied among the studies, and the definition of aspiration pneumonia was not standardized across the studies. We intended to include exposure data from both VFSS and FEES for the evaluation of swallowing function; however, none of the included studies evaluated swallowing function with FEES, which is a limitation of this review. Lastly, the included studies did not report data that could be used for any pre-determined subgroup analyses; therefore, we could not comment on whether the risk of aspiration pneumonia would be different based on age; depth of laryngeal penetration; type of intervention, such as thickening of feeds; and comorbidities, such as a neurological or syndromic diagnosis. 

### 4.4. Implication for Practice

The evaluation of oropharyngeal dysphagia is complex, and instrumental swallowing evaluation using VFSS or FEES is essential for the diagnostic process. This systematic review demonstrated that the odds of aspiration pneumonia were lower with laryngeal penetration than with tracheal aspiration. However, the significance of laryngeal penetration in VFSS needs to be interpreted in the context of other clinical findings, such as the presence of a neurological disorder, history of prematurity, known anatomical malformations of the oropharynx, and history of surgery. The association between aspiration pneumonia and laryngeal penetration is probably small, being without any major risk factors for swallowing dysfunction. However, in children at high risk of aspiration pneumonia, these findings need to be interpreted carefully as VFSS is a brief study, and the presence of laryngeal penetration may be a harbinger of missed aspiration. 

### 4.5. Implication for Research

Future studies with larger sample sizes are needed to establish an association between laryngeal penetration and aspiration pneumonia. Future studies should use a standard definition for laryngeal penetration and aspiration pneumonia. Clinically important outcomes, such as PICU admission, hospitalization, symptoms, and mortality, should be reported in future studies. Data for subgroup analysis based on age, CNS morbidities, syndromic diagnosis, and anatomic anomalies should be reported to assess whether the risk of aspiration pneumonia varies across these subgroups. Furthermore, the risk of aspiration pneumonia should be investigated based on the depth of laryngeal penetration, and better definitions of landmarks for laryngeal penetration spanning the borders of the larynx and supraglottic larynx should be pursued.

## 5. Conclusions

In pediatric patients with dysphagia, the presence of tracheal aspiration in VFSS demonstrated increased odds for aspiration pneumonia, though the odds were lower in laryngeal penetration for the same association. The included studies’ quality varied, and several clinically important outcomes were not reported. Future studies with large sample sizes and standardized definitions of exposure and outcome are needed to determine the clinical significance of laryngeal penetration and tracheal aspiration in video-fluoroscopic studies in children.

## Figures and Tables

**Table 1:** Characteristics of included studies.

Author	Type of Study	Country	Number of Participants	Inclusion Criteria	Definition of Laryngeal Penetration	Definition of Aspiration	Definition of Aspiration Pneumonia
Coon 2016 [25]	Retrospective cohort	USA	576	Children with difficulty swallowing during infancy	Not clearly described	Not clearly described	Diagnosed based on ICD-9A coding
Gurberg 2015 [11]	Retrospective case control	Canada	107	Patients referred to a swallowing and dysphagia clinic with no extra-laryngeal etiologies of pneumonia	Passage of material into the laryngeal vestibule above the true vocal folds	Entry of bolus material below the true vocal folds	Clinical diagnosis of pneumonia made by a physician, along with consolidation via chest x-ray
Kemps 2015 [12]	Retrospective cohort	Canada	205	Pediatric patients referred to a swallowing and dysphagia clinic	Passage of contrast material into the laryngeal vestibule above the vocal cords	Passage below the vocal cords	Clinical diagnosis made by a physician, along with confirmation via chest x-ray
Kim 2014 [26]	Retrospective cohort	USA	30	Random sample of patients who were referred to a dysphagia clinic	Supraglottic penetration	Subglottic aspiration	As assessed by clinician
Kim 2018 [27]	Retrospective cohort	South Korea	110	Pediatric patients with dysphagia who underwent both VFSS and salivagram	Food material entered the airway above the vocal folds	Entery of food material below vocal folds,	Clinical findings, infiltration via chest X-ray, and systemic inflammation, based on laboratory findings and absence of pathogens causing atypical pneumonia.
Lefton-Greif 2006 [28]	Retrospective cohort	USA	19	Patients referred to speech–language pathology for swallowing evaluations using VFSS	Contrast entering the level of the vocal folds, but not below the vocal cords	Contrast entering into the subglottic airway or trachea	Definition not clearly described
McSweeney 2020 [29]	Retrospective cohort	USA	1668	Infants and children <2 years with symptoms consistent with aspiration on VFSS	As defined by speech pathologist	As defined by speech pathologist	Outcome not reported
Miranda 2022 [30]	Retrospective cohort	Brazil	164	Infants aged between 0 and 12 months who underwent VFSS during hospital admission	Used Rosenbek’s penetration–aspiration scale	Used Rosenbek’s penetration–aspiration scale	Outcome not reported
Newman 2001 [13]	Retrospective cohort	USA	43	Infants referred for VFSS	Material entering the vestibule or entrance of the airway and traveling to any extent down toward the level of the true vocal folds	Passage of material below the vocal cords	As defined by clinician
Pavithran 2019 [31]	Retrospective case control	India	35	The children aged 0 to 10 years with dysphagia who underwent VFSS	Contrast reaching the level the vocal cords	Contrast reaching the subglottis	Fever, moist cough, tachypnea, and dyspnea
Taniguchi 1994 [6]	Retrospective case control	USA	142	All pediatric patients who underwent VFFS	Entry of material into the airway, but not passing the true vocal cords	Entry of material below the true vocal folds	Pneumonia caused by the aspiration of oral or gastric contents
Weir 2007 [7]	Retrospective case control	Australia	135	Children who underwent VFSS	Entry of material into the laryngeal vestibule, but not passing below the true vocal folds	The passage of material below the level of the true vocal folds	WHO definition: cough, fever, tachypnea, and dyspnea
Wier 2011 [32]	Prospective cohort	Australia	102	Children undergoing VFSS	Used 8-point penetration–aspiration scale	Recurrent aspiration of saliva, food, and/or fluids below the level of the vocal folds	WHO definition: cough, fever, tachypnea, and dyspnea

ICD, International Classification of Disease.

## Data Availability

We report most of the data in tables and figures. Additional details are available on request from the corresponding author.

## Supplementary Materials

The following supporting information can be downloaded at: https://www.mdpi.com/article/10.3390/jcm12124087/s1, Table S1: Excluded studies and exclusion reasons Table S2: ROBINS risk of bias assessment with explanations Table S3: GRADE evidence profile: Laryngeal penetration compared to No laryngeal penetration in pediatric patients with dysphagia Table S4: GRADE evidence profile: Tracheal aspiration vs. no tracheal aspiration in pediatric patients with dysphagia.
