# Supplementary material for: Laryngeal Penetration and Risk of Aspiration Pneumonia in Children with Dysphagia—A Systematic Review
Source: J Clin Med. 2023 Jun 16;12(12):4087. doi: 10.3390/jcm12124087 (PMC10299101; doi:10.3390/jcm12124087)
Supplement: Supplementary file 1 [file jcm-12-04087-s001.zip › jcm-2434219-supplementary.pdf]

Table S1. Excluded studies and exclusion reasons

Author	Year of Publication	Reason for Exclusion
Serel, S[1]	2013	Lack of Outcomes
Erdur, O[2]	2017	No Comparison Group
Kim, B.R[3]	2010	Ineligible Study Design
Levy, D.S[4]	2019	Lack of Outcomes
Rosen, R[5]	2018	Lack of Outcomes
Duncan, D[6]	2018	No Comparison Group
Kyoung-Moo, L. E. E[7]	2017	Lack of Outcomes
Schwarz, S. M[8]	2001	Adult population
Bock, J.M[9]	2017	Adult Population
Shin, J.C[10]	2011	Adult population
Wong, N.S[11]	2019	Adult population
Lagos-Guimarães, H. N. C[12]	2016	Adult population
Lefton-Greif, M. A[13]	2000	Adult population
Alnassar, M[14]	2011	Ineligible Intervention
Duncan, D.R[15]	2018	Ineligible Intervention
Bowman, O. Jayne[16]	2020	Lack of Outcomes
Osborn, A. J[5]	2014	Adult population
Stanley, M. A[16]	2019	Adult population
Teive, H. G.[17]	2014	Adult population
Terré, R.[16]	2009	Adult population
Schurr, M. J[16]	1999	Adult population
Madhoun, L[18]	2018	Adult population
Morgan, A[16]	2005	Adult population
Birutis, R.I[16]	2013	Adult population

Soria, F[19]	2013	Adult population
Rosen, R[20]	2018	Duplicate Study
Lee, J. H[21]	2011	Lack of Outcomes
De Fatima Da Silva Munhoz, L[22]	2015	Lack of Outcomes
Kyeong-Woo, Lee[16]	2011	Lack of Outcomes
Duncan, D[23]	2017	Lack of Outcomes
Dharmarathna, I[24]	2021	Lack of Outcomes
Diniz, P[25]	2015	Duplicate Study
Akel, K[11]	2017	Ineligible Study Design
Duncan, D.[12]	2018	Lack of Outcomes
Dharmarathna, Isuru[26]	2021	Ineligible Intervention
Diniz, P. B[27]	2017	Lack of Outcomes
Levy, D. S[28]	2018	Lack of Outcomes
Kyeong-Eun, U. H. M[29]	2013	Lack of Outcomes
Kaymaz, Nazan[30]	2017	Ineligible Study Design

Table S2. ROBINS risk of bias assessment with explanations

Author	Year	Outcome Studied	Overall Risk of Bias Assessment	Reason for Risk of Bias
Maireade E. McSweeney [13]	2020	Enteral Feeding	High	(1) Did not account for all confounders (2) Did not account for time-varying confounders
Pavithran, Jayanthy [31]	2019	Aspiration Pneumonia	Moderate	Potential for confounding effects
Kim, GE [6]	2018	Aspiration Pneumonia	High	(1) Did not account for all confounders (2) Did not account for time-varying confounders
Kim, Bo Ryun [7]	2014	Enteral Feeding	Moderate	Potential for confounding effects
Gurberg, Joshua [32]	2015	Aspiration Pneumonia	High	(1) Did not account for all confounders (2) Did not account for time-varying confounders (3) Outcome data unavailable for all participants (4) Participants excluded due to missing data for analysis
Kemps, Glen [25]	2015	Aspiration Pneumonia	Moderate	(1) Potential for confounding effects (2) Participants excluded due to missing data for analysis

Newman, Lisa A.[26]	2001	Aspiration Pneumonia	High	(1) Did not account for all confounders (2) Did not account for time-varying confounders (3) Intervention groups not clearly defined
Lefton-Greif, Maureen A. [27]	2006	Aspiration Pneumonia	High	(1) Did not account for all confounders (2) Did not account for time-varying confounders (3) Intervention groups not clearly defined (4) Participants excluded due to missing data for analysis (5) Reasons for missing data inconsistent among all interventions (6) Lack of evidence that results were robust to the presence of missing data
Lefton-Greif, Maureen A. [28]	2006	Enteral Feeding	High	(1) Did not account for all confounders (2) Did not account for time-varying confounders (3) Intervention groups not clearly defined (4) Participants excluded due to missing data for analysis (5) Reasons for missing data inconsistent among all interventions (6) Lack of evidence that results were robust to the presence of missing data
Weir, Kelly [29]	2011	Aspiration Pneumonia	Moderate	Potential for confounding effects
Weir, Kelly [27]	2011	Enteral Feeding	Moderate	Potential for confounding effects
Taniguchi, M. H [30]	1994	Aspiration Pneumonia	Moderate	(1) Potential for confounding effects (2) Participants excluded due to missing data for analysis (3) Lack of evidence that results were robust to the presence of missing data
Weir, Kelly [13]	2007	Aspiration Pneumonia	Moderate	Potential for confounding effects

Footnote: Risk of bias was assessed according to Cochrane risk of bias tool for observational studies (ROBINs-1). We assessed the risk of bias from each study for a given outcome.

Supplementary Table S3: GRADE evidence profile

Question: Laryngeal penetration compared to No laryngeal penetration in pediatric patients with dysphagia
Setting: mainly outpatient

Certainty assessment							№ of patients		Effect	Certainty
№ of studies	Study design	Risk of bias	Inconsistency	Indirectness	Imprecision	Other considerations	Laryngeal penetration	No laryngeal penetration	Relative (95% CI)	
**Aspiration Pneumonia**										
6	observational studies	serious[a]	not serious	not serious	serious[b]	none	294	251	**OR 1.44** (0.94 to 2.19)	⨁⨁◯◯ Low
**Enteral Tube**										
1	observational studies	serious[b]	not serious	not serious	serious[b]	none	66	46	**OR 0.77** (0.26 to 2.29)	⨁⨁◯◯ Low

Abbreviations: CI: confidence interval; **OR:** odds ratio

Explanations

a. Two of the included studies were at high risk of bias because they did not adjust for any confounders.
b. The confidence interval around the summary estimate was wide and included null effects.

Table S4. GRADE evidence profile

Question: Tracheal aspiration compared to No tracheal aspiration in pediatric patients with dysphagia
Setting: mainly outpatient

Certainty assessment							№ of patients		Effect	Certainty
№ of studies	Study design	Risk of bias	Inconsistency	Indirectness	Imprecision	Other considerations	Tracheal Aspiration	No Tracheal Aspiration	Relative (95% CI)	
**Aspiration Pneumonia**										
7	observational studies	serious[a]	not serious	not serious	not serious	none	-/344	-/453	**OR 2.72** (1.86 to 3.98)	⨁⨁⨁◯ Moderate
**Enteral Tube**										
2	observational studies	serious	not serious	not serious	serious[b]	none	-/139	-/273	**OR 1.32** (0.58 to 2.98)	⨁⨁◯◯ Low
Hospitalization										
1	observational studies	not serious	not serious	not serious	not serious	none	-/0	-/0	**RR 1.043** (1.010 to 1.070)	⨁⨁⨁⨁ High

CI: confidence interval; **OR:** odds ratio; **RR:** risk ratio

Explanations

a. Two of the included studies were at high risk of bias because they did not adjust for any of the confounders.
b. The confidence interval around the summary estimate was wide and included a null effect.

References

1. Bhattacharyya N. The prevalence of pediatric voice and swallowing problems in the United States. *Laryngoscope* 2015; 125: 746-750. 2014/09/16. DOI: 10.1002/lary.24931.
2. Lawlor CM and Choi S. Diagnosis and Management of Pediatric Dysphagia: A Review. *JAMA Otolaryngol Head Neck Surg* 2020; 146: 183-191. DOI: 10.1001/jamaoto.2019.3622.
3. Covidence systematic review software, Veritas Health Innovation, Melbourne, Australia. Available at www.covidence.org.
4. Durvasula VS, O'Neill AC and Richter GT. Oropharyngeal Dysphagia in children: mechanism, source, and management. *Otolaryngol Clin North Am* 2014; 47: 691-720. 2014/09/13. DOI: 10.1016/j.otc.2014.06.004.
5. Rosenbek JC, Robbins JA, Roecker EB, et al. A penetration-aspiration scale. *Dysphagia* 1996; 11: 93-98. 1996/01/01. DOI: 10.1007/BF00417897.
6. Taniguchi MH and Moyer RS. Assessment of risk factors for pneumonia in dysphagic children: significance of videofluoroscopic swallowing evaluation. *Dev Med Child Neurol* 1994; 36: 495-502. 1994/06/01. DOI: 10.1111/j.1469-8749.1994.tb11879.x.
7. Weir K, McMahon S, Barry L, et al. Oropharyngeal aspiration and pneumonia in children. *Pediatr Pulmonol* 2007; 42: 1024-1031. 2007/09/26. DOI: 10.1002/ppul.20687.
8. Delzell PB, Kraus RA, Gaisie G, et al. Laryngeal penetration: a predictor of aspiration in infants? *Pediatr Radiol* 1999; 29: 762-765. 1999/10/20. DOI: 10.1007/s002470050690.
9. Friedman B and Frazier JB. Deep laryngeal penetration as a predictor of aspiration. *Dysphagia* 2000; 15: 153-158. 2000/06/06. DOI: 10.1007/s004550010018.
10. Serel Arslan S, Demir N and Karaduman AA. Both pharyngeal and esophageal phases of swallowing are associated with recurrent pneumonia in pediatric patients. *Clin Respir J* 2018; 12: 767-771. 2016/12/08. DOI: 10.1111/crj.12592.
11. Gurberg J, Birnbaum R and Daniel SJ. Laryngeal penetration on videofluoroscopic swallowing study is associated with increased pneumonia in children. *Int J Pediatr Otorhinolaryngol* 2015; 79: 1827-1830. 2015/09/19. DOI: 10.1016/j.ijporl.2015.08.016.
12. Kemps G, Sewitch M, Birnbaum R, et al. Contrast pooling in videofluoroscopic swallowing study as a risk factor for pneumonia in children with dysphagia. *Int J Pediatr Otorhinolaryngol* 2015; 79: 1306-1309. 2015/06/21. DOI: 10.1016/j.ijporl.2015.05.039.
13. Newman LA, Keckley C, Petersen MC, et al. Swallowing function and medical diagnoses in infants suspected of Dysphagia. *Pediatrics* 2001; 108: E106. 2001/12/04. DOI: 10.1542/peds.108.6.e106.
14. Duncan DR, Amirault J, Mitchell PD, et al. Oropharyngeal Dysphagia Is Strongly Correlated With Apparent Life-Threatening Events. *J Pediatr Gastroenterol Nutr* 2017; 65: 168-172. 2016/10/16. DOI: 10.1097/MPG.0000000000001439.
15. Page MJ, McKenzie JE, Bossuyt PM, et al. The PRISMA 2020 statement: an updated guideline for reporting systematic reviews. *BMJ* 2021; 372: n71. 2021/03/31. DOI: 10.1136/bmj.n71.

16. Adlakha V, Ramos L, Smith A, et al. Risk of aspiration pneumonia in paediatric patients with dysphagia who were found to have laryngeal penetration on the instrumental swallow evaluation: a systematic review protocol. *BMJ Open* 2021; 11: e048422. 2021/08/05. DOI: 10.1136/bmjopen-2020-048422.
17. WHO classification and treatment of pneumonia in children at health facilities: evidence summaries. [Internet]. 2014 [cited 2020 Dec 21]. Available from: .
18. Covidence systematic review software VHI, Melbourne, Australia. Available at www.covidence.org.
19. Sterne JA, Hernan MA, Reeves BC, et al. ROBINS-I: a tool for assessing risk of bias in non-randomised studies of interventions. *BMJ* 2016; 355: i4919. 2016/10/14. DOI: 10.1136/bmj.i4919.
20. Review Manager (RevMan) [Computer program]. Version 5.3. Copenhagen: The Nordic Cochrane Centre TCC, 2014. .
21. Higgins JPT TJ, Chandler J, Cumpston M, Li T, Page MJ, Welch VA (editors). Cochrane Handbook for Systematic Reviews of Interventions version 6.1. *Cochrane* 2020.
22. Melsen WG, Bootsma MC, Rovers MM, et al. The effects of clinical and statistical heterogeneity on the predictive values of results from meta-analyses. *Clin Microbiol Infect* 2014; 20: 123-129. 2013/12/11. DOI: 10.1111/1469-0691.12494.
23. Guyatt G, Oxman AD, Akl EA, et al. GRADE guidelines: 1. Introduction-GRADE evidence profiles and summary of findings tables. *J Clin Epidemiol* 2011; 64: 383-394. 2011/01/05. DOI: 10.1016/j.jclinepi.2010.04.026.
24. GRADEpro. GRADEpro Guideline Development Tool [Software]. McMaster University and Evidence Prime, Available from gradepro.org, (2022).
25. Coon ER, Srivastava R, Stoddard GJ, et al. Infant Videofluoroscopic Swallow Study Testing, Swallowing Interventions, and Future Acute Respiratory Illness. *Hosp Pediatr* 2016; 6: 707-713. DOI: 10.1542/hpeds.2016-0049.
26. Kim BR, Sung IY, Choi KH, et al. Long-term outcomes in children with swallowing dysfunction. *Dev Neurorehabil* 2014; 17: 298-305. 20130719. DOI: 10.3109/17518423.2013.770102.
27. Kim GE, Sung IY, Ko EJ, et al. Comparison of Videofluoroscopic Swallowing Study and Radionuclide Salivagram for Aspiration Pneumonia in Children With Swallowing Difficulty. *Ann Rehabil Med* 2018; 42: 52-58. 20180228. DOI: 10.5535/arm.2018.42.1.52.
28. Lefton-Greif MA, Carroll JL and Loughlin GM. Long-term follow-up of oropharyngeal dysphagia in children without apparent risk factors. *Pediatr Pulmonol* 2006; 41: 1040-1048. DOI: 10.1002/ppul.20488.
29. McSweeney ME, Meleedy-Rey P, Kerr J, et al. A Quality Improvement Initiative to Reduce Gastrostomy Tube Placement in Aspirating Patients. *Pediatrics* 2020; 145. DOI: 10.1542/peds.2019-0325.
30. Miranda PP, Levy DS and Kieling RR. Aspiration in the First Year of Life and Later Tube Feeding: A Retrospective Cohort from a Low-Income Country. *Dysphagia* 2022 20220427. DOI: 10.1007/s00455-022-10450-4.
31. Pavithran J, Puthiyottil IV, Narayan M, et al. Observations from a pediatric dysphagia clinic: Characteristics of children at risk of aspiration pneumonia. *Laryngoscope* 2019; 129: 2614-2618. 20181224. DOI: 10.1002/lary.27654.
32. Weir KA, McMahon S, Taylor S, et al. Oropharyngeal aspiration and silent aspiration in children. *Chest* 2011; 140: 589-597. 20110324. DOI: 10.1378/chest.10-1618.
